# Imitating the respiratory activity of the brain stem by using artificial neural networks: exploratory study on an animal model of lactic acidosis and proof of concept

**DOI:** 10.1007/s10877-024-01208-4

**Published:** 2024-08-20

**Authors:** Gaetano Perchiazzi, Rafael Kawati, Mariangela Pellegrini, Jasmine Liangpansakul, Roberto Colella, Paolo Bollella, Pramod Rangaiah, Annamaria Cannone, Deepthi Hulithala Venkataramana, Mauricio Perez, Sebastiano Stramaglia, Luisa Torsi, Roberto Bellotti, Robin Augustine

**Affiliations:** 1 The Hedenstierna Laboratory, Department of Surgical Sciences, Uppsala University, Uppsala, Sweden; 2 Department of Anesthesia, Operation and Intensive Care, Uppsala University Hospital, Uppsala, Sweden; 3 Ministry of Education and Merit, Rome, Italy; 4 Department of Chemistry, University of Bari Aldo Moro, Bari, Italy; 5 Department of Electrical Engineering, Solid-State Electronics, Uppsala University, Uppsala, Sweden; 6 Department of Anaesthesia and Intensive Care, “Madonna delle Grazie” Hospital, Matera, Italy; 7 Department of Information Technology, Uppsala University, Uppsala, Sweden; 8 Dipartimento Interateneo di Fisica, Università degli Studi di Bari, Rome, Italy; 9 Istituto Nazionale di Fisica Nucleare, Sezione di Bari, Italy; 10 Hedenstierna Laboratoriet, Akademiska sjukhuset ing 40 3 tr, Uppsala, 75185 Sweden

**Keywords:** Artificial neural networks, Lactic acidosis, Closed loop, Mechanical ventilation, Translational model

## Abstract

Artificial neural networks (ANNs) are versatile tools capable of learning without prior knowledge. This study aims to evaluate whether ANN can calculate minute volume during spontaneous breathing after being trained using data from an animal model of metabolic acidosis. Data was collected from ten anesthetized, spontaneously breathing pigs divided randomly into two groups, one without dead space and the other with dead space at the beginning of the experiment. Each group underwent two equal sequences of pH lowering with pre-defined targets by continuous infusion of lactic acid. The inputs to ANNs were pH, ΔPaCO_2_ (variation of the arterial partial pressure of CO_2_), PaO_2_, and blood temperature which were sampled from the animal model. The output was the delta minute volume (ΔV_M_), (the change of minute volume as compared to the minute volume the animal had at the beginning of the experiment). The ANN performance was analyzed using mean squared error (MSE), linear regression, and the Bland-Altman (B-A) method. The animal experiment provided the necessary data to train the ANN. The best architecture of ANN had 17 intermediate neurons; the best performance of the finally trained ANN had a linear regression with R^2^ of 0.99, an MSE of 0.001 [L/min], a B-A analysis with bias ± standard deviation of 0.006 ± 0.039 [L/min]. ANNs can accurately estimate ΔV_M_ using the same information that arrives at the respiratory centers. This performance makes them a promising component for the future development of closed-loop artificial ventilators.

**Supplementary Information:**

The online version contains supplementary material available at 10.1007/s10877-024-01208-4.

## Introduction

The main function of the respiratory system is to secure gas exchange between the tissues of the body and the external environment, by this way contributing to acid-base homeostasis by regulating CO_2_ elimination [1]. The respiratory system comprises several functional components corresponding to distinct anatomical structures: a central neural control, a sensory input system, and an effector apparatus. The central neural control (mainly composed of centers located in the pons and the medulla) integrates information arriving from the cortex and a series of receptors (peripheral and central chemoreceptors, mechanoreceptors, and metaboreceptors) and conveys signals to respiratory muscles (diaphragm and accessory muscles of respiration and upper airway muscles) and upper airway (for the reflexes of airway protection and patency) [2].

The respiratory centers adjust not only the amount of ventilation (i.e., minute volume) but also, by integrating information from the peripheral mechanoreceptors, decide the best pattern of ventilation in terms of frequency and tidal volume (V_T_) [3] that should also correspond to a minimum possible work of breathing [4]. From this simple description is clear that the system is based on different informative flows that have been the object of many studies [5] and different deterministic models of its function have been developed [6].

A relevant field of study in critical care medicine is mechanical ventilation which aims to support a failing respiratory function, trying to mimic the respiratory system. For the above-mentioned difficulties in continuously quantifying the components of the respiratory system, a standard method to implement mechanical ventilation is based on taking intermittently blood samples from a peripheral artery, measuring the blood gases, and manually adjusting the ventilator. The quest for creating tools able to support patient ventilation in closed-loop has brought to systems that combine in different ways information derived from the expired CO_2_ or the respiratory activity of the patient [7].

None of them is currently able to use simultaneous information coming from the real control points of the respiratory function, i.e., the partial pressure of the respiratory gases and the pH inside the arterial system.

Artificial neural networks (ANNs) are universal function approximators [8] and can reproduce the function of different systems, provided they are fed with the input and the output to and from these systems. One of the major problems in training an ANN (and in general all the data-driven models) when they have to imitate biological behaviors is the pooling of a sufficient amount of data able to cover all the possible spectrum of responses of the biological reality. On the other side, the use of non-biological models to generate tracings to be forwarded to an ANN, although useful, has as a major limitation that a perfectly trained ANN will learn the implemented model and may not map the complex interrelationships typical of a biological structure [9].

The main strategy to which this exploratory study belongs, is to propose a method to create a map of the physiological interaction between ventilation and arterial blood gases to be used for training ANNs capable to control an artificial ventilator.

This specific experiment aims to determine whether a neural network, trained on data deriving from signals afferent to the respiratory centers, can replicate the same response as these centers.

Specifically, we aimed to train an ANN using the partial pressures of respiratory gases and blood pH, observed during spontaneous ventilation in various conditions, such as the induction of metabolic acidosis and the application of a respiratory dead space. Successively we estimated the performance of the trained ANN in mimicking the biological respiratory response.

The aim of this contribution is to establish a proof of concept for training Artificial Neural Networks to imitate the behavior of physiological mechanisms when data collection is limited due to the infrequency of the condition to be reproduced. To address this issue, potential solutions may be offered by utilizing translational models.

## Materials and methods

### Anesthesia and preparation

This study was approved by the Local Animal Ethics Committee of Uppsala University.

Data were collected from ten healthy pigs (Yorkshire, Hampshire and Swedish breeds) with weight of 29.1 ± 2.9 Kg (mean ± standard deviation). The animals were anesthetized with medetomidine 5 mg/Kg (Dormitor Vet., Orion Pharma, Sollentuna Sweden), tiletamine zolazepam 5 mg/Kg (Zoletil, Boehringer Ingelheim, Copenhagen, Denmark) and fentanyl 5 µg/Kg/h (Pharmalink, Spanga, Sweden). Anesthesia was maintained by a continuous infusion of ketamine (Ketaminol, Vetpharma AB, Zurich, Switzerland), diluted in Rehydrex with glucose (Fresenius Kabi Uppsala, Sweden).

During instrumentation, succinylcholine (Celocurinklorid, NM Pharma, Stockholm, Sweden) was administered for myorisolution, first in bolus and then by continuous infusion of 1 mg/ml in glucose 5%.

The animals were intubated (tube n.7, Hi-Contour, Mallinckrodt Medical, Athlone; Ireland). Two 20-gauge catheters were inserted into both femoral arteries: one for arterial pressure monitoring and the other to insert the blood gas analysis sensor (see details below).

A Swan-Ganz catheter was placed in the internal jugular vein, surgically isolated, to administer drugs in the central vein and to monitor on a screen (CS/3 TM, Datex Ohmeda, Helsinki, Finland) the arterial and capillary pulmonary pressure. The cardiac output was estimated by the thermodilution method, by injecting cold boluses of a glucose solution in triplicate (10 ml, at 3–5 °C), randomly during the respiratory cycle.

During the surgical procedures, the pigs were ventilated in volume controlled constant flow mode (VC-CFMV), starting with a tidal volume of 8 ml/Kg and progressively titrated to obtain normocapnia. The inspiration/expiration ratio was 1:2 (s), the fraction of inspired oxygen (F_I_O_2_ ) was 0.5, and the positive end-expiratory pressure (PEEP) was 5 cmH_2_O.

Airway pressure and flow were recorded by a D-Lite connector (Datex Ohmeda, Helsinki, Finland), mounted on the endotracheal tube. Esophageal and gastric pressures were measured by dedicated esophageal balloon catheters (Erich Jaeger GmbH, Höchberg, Germany). The D-Lite connector ports and the balloon catheters were connected to differential pressure transducers (Sensym, Sensor Technics, Pucheim, Germany) which were calibrated using a water column before each experimental session. Data were collected by using purposely written scripts for the Labview data acquisition system (LabView, National Instruments, Austin, TX, USA).

Blood gas parameters were continuously measured using miniaturized hybrid probes that employed optode technology for pH and the partial pressure of carbon dioxide in arterial blood (PaCO_2_), a miniaturized Clark electrode for the partial pressure of oxygen in the arterial blood (PaO_2_), and a thermocouple for measuring temperature [10]. These sensors were assembled in a probe with a diameter of 0.5 mm, which could be easily inserted into a 20 G arterial cannula. The probes were then connected to the Paratrend blood gas monitoring system.(Diametrics Medical, High Wycombe, England) which also yielded the calculated concentration of HCO_3_^−^, Base Excess (BE) and arterial oxygen saturation (S_a_O_2_). Data from the Paratrend monitor were continuously sampled at 0.5 Hz and recorded on a connected laptop through a serial port.

Urinary output was estimated by a catheter, positioned in a laparotomic way. During the entire experiment, the animals were covered by a servo-controlled thermic blanket to ensure normothermia. The surgical wounds deriving from preparation were infiltrated with xylocaine to limit the potential pain, which could, in turn, raise the animal’s respiratory frequency.

After the preparation (anesthesia, intubation, surgery and insertion of catheters), the animals were weaned from mechanical ventilation by reducing ketamine (from 21.7 mg/kg/h to 11.8 mg/kg/h) and ceasing the infusion of succinylcholine: by this way the pigs regained spontaneous breathing while simultaneously maintained sedation and analgesia. Once reached this point, after thirty minutes of stabilization, the animals could be disconnected from the ventilator and the experimental protocol started.

### Experimental protocol

**Fig. 1:**
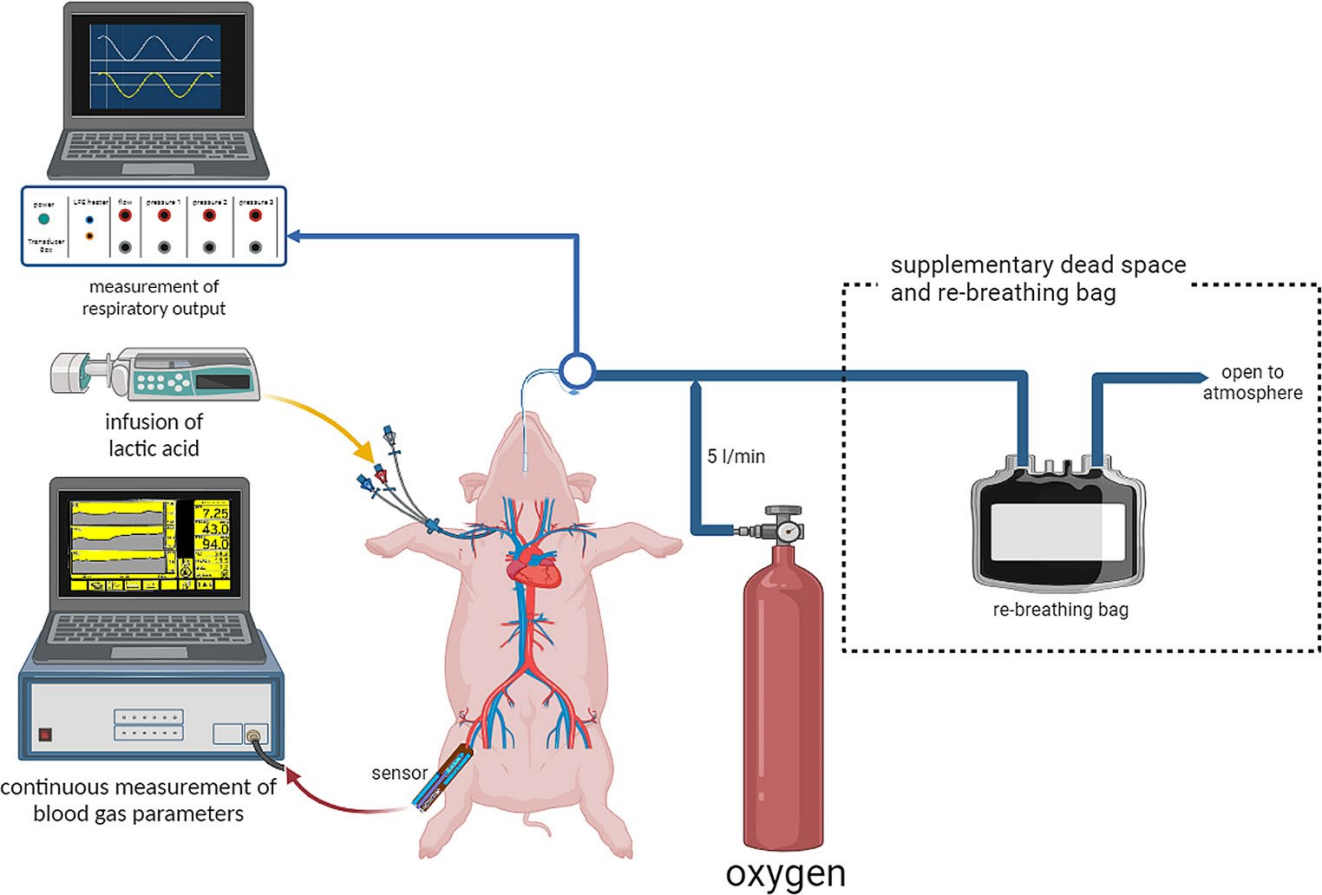
Experimental setup (*created with BioRender.com*)

**Fig. 2:**
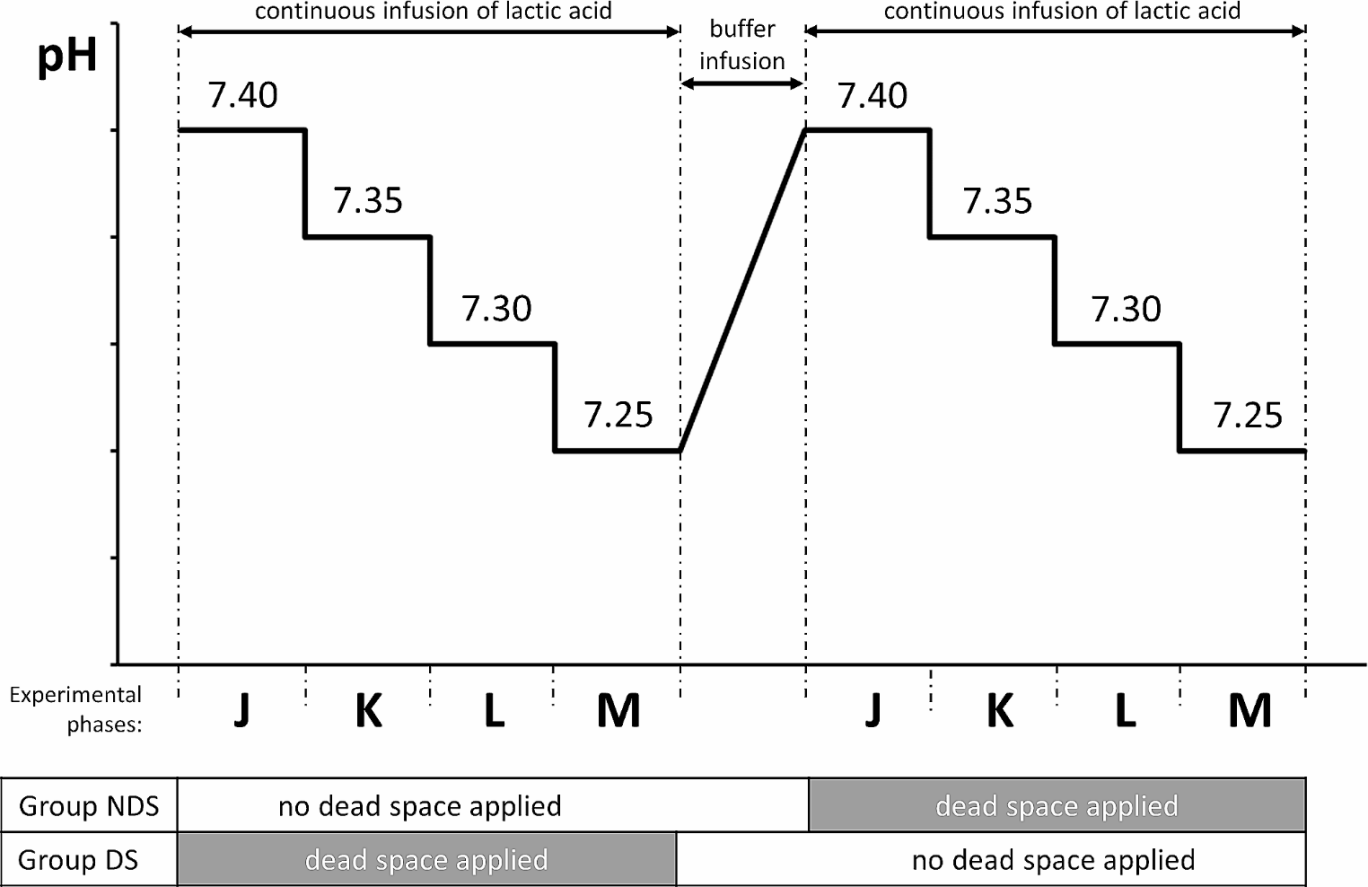
Outline of the physiological experiment

The time course of each experiment was divided into two equal sequences of pH lowering, differing in the timing of application of an additional external dead space, consisting of a connected rebreathing bag (henceforth abbreviated as *dead space*, see Figs. 1 and 2).

In both phases, the blood pH was steered by continuously administering an infusion of lactic acid 0,9 M (Riedel-De Haen, RdH Laborchemikalien GmbH & Co, Seelze, Germany.

by a pump-syringe in the central vein, according to Shirer’s protocol [11] and starting with an infusion dose of 0.08 ml/kg/min.

The objective was to reduce the blood pH in a controlled fashion, reaching the pre-defined targets of pH 7.40, 7.35, 7.30, and 7.25 while measuring the above-mentioned respiratory and arterial blood gas parameters. To achieve these goals and have the actual pH on the screen, the infusion speed could be slowly adjusted to reach these targeted steady-state pHs. A continuous supply of Oxygen of 5 L/min was provided at the tip of the endotracheal tube.

The pigs were divided randomly into two groups, which differed in the timing of the application of the mentioned additional dead space. This last consisted of a modified reservoir Tedlar bag of 200 L (Hans Rudolph, Wyandotte, Kansas City, USA) connected to the endotracheal tube and open on the distal side to the atmosphere. through which the animal had to breathe.

We hypothesized that when the blood pH changes and dead space is introduced, animals would naturally adjust their breathing pattern to establish respiratory compensation, aiming to restore the pH to normal physiological levels.

The two groups were labeled in relation to the application of dead space at the beginning of the experiment, thus obtaining the NDS group (= No Dead Space, characterized by the absence of dead space at the beginning of the experiment) and the DS group (having dead space at the beginning of the experiment).

After the series of measurements was completed at pH 7.25, lactic acid infusion was interrupted and pH was progressively normalized using 1000 ml of normal saline and the buffer Tribonat (Pharmacia AB, Stockholm, Sweden), following the changes of pH on the screen. After the pH returned to 7.40 and a further twenty minutes of stabilization, the following step was a respiratory dead space modification.

Administering lactic acid again via an infusion pump, the pH was changed to 7.35, 7.30, and 7.25, and, at each of these pH values, the same hemodynamic, blood gas, and respiratory variables were measured (see Fig. 2).

During each pH step, we recorded the mentioned respiratory parameters for 10 s, corresponding to 5 samples per step. In summary, considering 10 pigs, 4 pH levels, 2 dead space statuses (on and off), and 5 samples per condition, we planned to pool 400 patterns to be forwarded to the ANNs.

At the end of the experiments, the pigs were euthanized during general anesthesia with a high dose of potassium chloride given intravenously.

### Artificial neural networks

The ANNs were implemented via software on a computer (MatLab ver 2021b, MathWorks, Natick, MA). In the ANNs utilized in this study, every neuron in the previous layer is connected to each neuron in the subsequent layer. These connections between neurons are assigned specific weights, and adjusting their values enables the network to associate input patterns with the correct output. Each neuron sums all the inputs arriving on itself from the preceding layers, and if the weighted sum of the afferent stimuli reaches a threshold (like biological neurons), it discharges downwards. So, once the training process is concluded, and a pattern is fed into the ANN, its neurons interact in a feed-forward manner, eventually producing the desired output.

The training function updating the ANN weights was a Bayesian regularization backpropagation. The input layer of the network was defined by the number of arterial blood gas (ABG) analysis parameters that were given simultaneously; they were four: pH, ΔPaCO_2_, PaO_2_, and temperature in the blood (T). ΔPaCO_2_ corresponds to the variation of the partial pressure of CO_2_ compared to the CO_2_ that the animal shows at the beginning of the experiment before the change of pH. The output layer was composed of one neuron, yielding the ΔV_M_, namely the change of minute volume as compared to the V_M_ the animal had at the beginning of the experiment. The ANN structure was a classical function approximator (a multilayer perceptron with a single hidden layer) characterized by a sigmoid transfer function in the hidden layer and a linear transfer function in the output layer.

The training was based on giving at the same time the pattern of blood gas analysis (pH, ΔPaCO_2_, PaO_2_, T) and simultaneously the value of ΔV_M_ that the animal presents. The learning function updates the weights of the neurons in order to make the ANN infer the relation between input and output. During the testing phase of the ANN only the ABG parameters are given, and the ANN yields its estimation of ΔV_M_. Comparing the calculation of ΔV_M_ by the ANN and the real ΔV_M_ (measured during the experiment in the animal) was possible to compute the error in ANN estimation.

A series of preliminary tests were executed to determine the best ANN architecture, i.e., the number of intermediate neurons that provided the best performance for the required task. They consisted of training, validating, and testing ANN architectures differing in the number of intermediate neurons, ranging from 4 to 20. The patterns of input and output data were given in random order and did not follow the time sequence of their recording during the experiment.

Each architecture’s training was repeated five times, each starting with a random assignation of weights, and the network’s performance as mean squared error (MSE) was annotated.

In the end, it was possible to obtain the average MSE over the five tests of each architecture and realize which number of intermediate neurons provided the best performance.

The final training phase was started once the best ANN architecture was identified. The entire pool of available data was divided randomly into three groups: the training, the validation, and the test sets (respectively 70%, 15%, and 15% of the entire pool).

During this phase, 20 different ANNs, all sharing the same architecture (chosen during the previous phase) but differing in the initial random assignment of node weights, were trained. The training process stopped based on three criteria: (a) if the lowest MSE, as determined by the best ANN architecture identified in the preceding phase, was achieved; (b) If the number of iterations (indicating how many cycles the training data goes through) exceeded 1000; (c) if the MSE started to increase, indicating the need for early stopping of the training. The ANN model with the lowest MSE on the final test pool was selected as the eligible model for the following step, consisting of a “prospective” evaluation of the ANN: it consisted in assessing the performance of the ANN on new data, which were not seen before during the training.

### Statistical analysis

For the numerosity and the characteristics of the sample, no inference could be made about the normality of their distribution. For this reason, all the hemodynamic and respiratory samples were analyzed using the Wilcoxon two-sided signed rank test with a level  α= 0.05 and applying the Bonferroni correction for multiple comparisons. For each variable, we compared whether there was any statistically significant difference at the different pH levels and with and without the application of external dead space.

The performance of the ANN was measured in terms of MSE, linear regression (between the ΔV_M_ yielded by the ANN and the ΔV_M_ effectively measured during the experiment). The measurement error was studied according to the method of Bland and Altman [12]. Moreover, linear regression was also used to analyze whether the error by the ANN was related to the absolute level of ΔV_M_.

## Results

All the animals survived the experimental protocol.

The total number of collected patterns was 380 out of 400, with 20 not collected due to technical reasons.

### Physiological variables

**Fig. 3:**
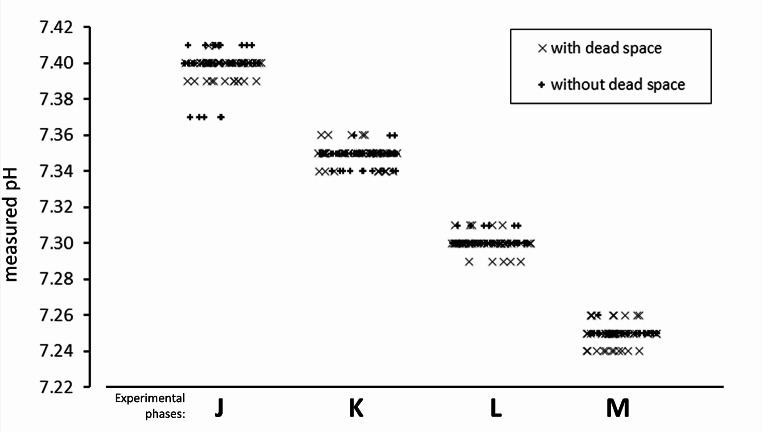
Individual effective pH values measured in the four experimental phases J, K, L, and M corresponding to imposed pH of 7.40, 7.35, 7.30, and 7.25, respectively

**Fig. 4:**
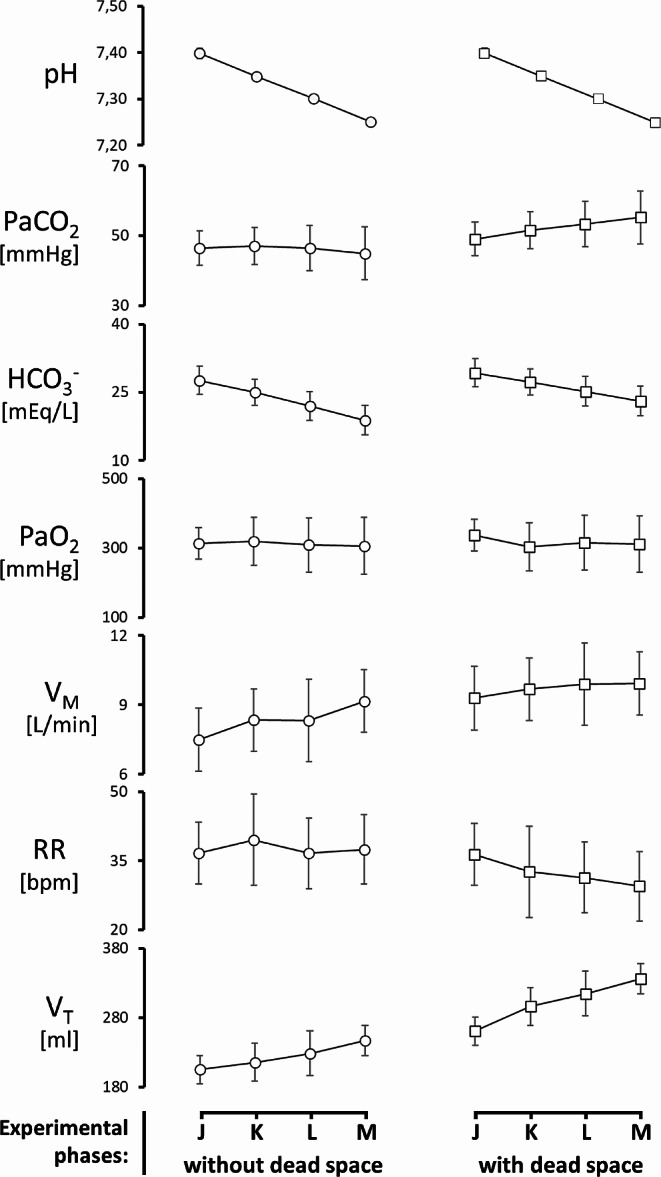
Representation of the main respiratory variables measured during the experiment. Data are reported as mean ± standard deviation. Abbreviations: PaCO_2_: partial pressure of carbon dioxide in arterial blood; HCO ^−^ _3_ : bicarbonate ion; PaO_2_: partial pressure of oxygen in arterial blood; V_M_: minute volume; RR: respiratory rate; V_T_: tidal volume; letters J, K, L, and M correspond to imposed pH of 7.40, 7.35, 7.30, and 7.25, respectively

The protocol of controlled reduction of pH (Fig. 3), with and without the application of dead space and rebreathing bag, induced different changes in the measured physiological variables as a consequence of compensatory mechanisms. A thorough description of their values is reported in Table 1 as well as in *online resource: supplementary Tables* from 1 A to 1D for their statistical analysis; Fig. 4 depicts the main physiological variables using time-synchronized coordinates.

It is worth noting that the measured pH was statistically different in the various steps of the experiment and did not differ when applying the external dead space.

The levels of PaCO_2_ were stable in the absence of applied dead space but tended to rise during the application of dead space, passing from 49.1 ± 7.8 [mmHg] at pH 7.40 to 55,2 ± 10,6 [mmHg] at pH 7.25. More specifically there was a statistically significant difference in PaCO_2_ at all pH levels below 7.40 when comparing measurements performed with and without applied dead space.

On the other side the HCO_3_^−^ was subjected to statistically significant changes both during the imposed step reduction of pH and also when comparing its values with and without the application of dead space, being the only exception at pH 7.40. The excess of bases followed the same pattern.

**Table 1:** Summary of physiological parameters measured during the experiment. Data are reported as mean ± standard deviation. Abbreviations: PaCO_2_: partial pressure of carbon dioxide in arterial blood; PaO_2_: partial pressure of oxygen in arterial blood; temp: temperature; HCO^−^ _3_ : bicarbonate ion; BE: base excess; SaO_2_ : oxygen saturation in arterial blood; V_M_: minute volume; V_T_ : tidal volume; RR: respiratory rate; S-SBP: systemic systolic blood pressure; S-DBP: systemic diastolic blood pressure; CO: Cardiac output; PCWP: pulmonary capillary wedge pressure; E_T_CO_2_ : end-tidal carbon dioxide; HR: heart rate. Letters J, K, L, and M correspond to imposed pH of 7.40, 7.35, 7.30, and 7.25, respectively

		DEAD SPACE OFF				DEAD SPACE ON			
variable	units	J	K	L	M	J	K	L	M
pH		7.40 ± 0.011	7.35 ± 0.006	7.30 ± 0.003	7.25 ± 0.003	7.40 ± 0.006	7.35 ± 0.003	7.30 ± 0.005	7.25 ± 0.006
PaCO_2_	[mmHg]	46.4 ± 4.9	47.0 ± 5.3	46.4 ± 6.5	45.0 ± 7.6	49.1 ± 7.8	51.6 ± 8.6	53.3 ± 10.0	55.2 ± 10.6
PaO_2_	[mmHg]	313.3 ± 46.0	320.1 ± 69.3	308.7 ± 78.9	306.3 ± 81.5	337.4 ± 33.9	303.7 ± 55.6	316.1 ± 56.8	311.2 ± 48.7
Temp	[°C]	38.4 ± 0.9	38.3 ± 0.9	38.3 ± 0.8	38.3 ± 0.8	38.3 ± 0.8	38.3 ± 0.8	38.2 ± 0.7	38.1 ± 0.7
HCO^−^ _3_	[mEq/L]	27.7 ± 3.1	25.0 ± 2.9	22.0 ± 3.2	18.9 ± 3.3	29.3 ± 4.7	27.3 ± 4.3	25.2 ± 4.7	23.1 ± 4.3
BE	[mEq/L]	3.4 ± 2.4	0.0 ± 2.2	-3.6 ± 2.4	-7.5 ± 2.6	4.6 ± 3.8	1.7 ± 3.4	-1.2 ± 3.7	-4.3 ± 3.3
SaO_2_	[%]	99.7 ± 0.1	99.6 ± 0.4	99.4 ± 0.9	99.3 ± 1.2	99.7 ± 0.1	99.6 ± 0.1	99.6 ± 0.1	99.6 ± 0.2
V_M_	[L/min]	7.5 ± 1.4	8.3 ± 1.4	8.3 ± 1.8	9.2 ± 1.4	9.3 ± 1.3	9.7 ± 1.6	9.9 ± 1.7	9.9 ± 1.5
V_T_	[ml]	205.3 ± 20.1	215.8 ± 27.4	228.5 ± 32.2	247.3 ± 21.7	260.5 ± 35.8	296.4 ± 34.7	314.8 ± 34.4	335.8 ± 21.9
RR	[bpm]	36.7 ± 6.8	39.6 ± 9.9	36.7 ± 7.7	37.5 ± 7.5	36.4 ± 7.8	32.6 ± 4.3	31.4 ± 3.5	29.5 ± 3.6
S-SBP	[mmHg]	115.8 ± 8.2	117.5 ± 12.7	122.4 ± 14.2	119.3 ± 12.4	114.9 ± 9.2	113.4 ± 8.9	116.7 ± 13.5	116.6 ± 15.5
S-DBP	[mmHg]	71.0 ± 9.9	72.6 ± 12.5	75.3 ± 14.5	62.9 ± 25.3	63.5 ± 13.4	63.6 ± 9.3	73.0 ± 21.3	70.9 ± 21.4
CO	[L/min]	4.7 ± 0.7	4.8 ± 0.9	5.2 ± 1.1	5.3 ± 0.9	5.6 ± 1.2	5.0 ± 1.0	5.3 ± 0.9	5.6 ± 0.9
PCWP	[mmHg]	4.3 ± 2.8	3.9 ± 2.0	3.7 ± 1.8	2.9 ± 0.9	3.4 ± 2.5	3.2 ± 2.7	2.4 ± 2.6	3.0 ± 2.4
E_T_CO_2_	[mmHg]	37.9 ± 4.5	36.7 ± 7.7	38.9 ± 9.5	35.5 ± 8.9	43.9 ± 7.4	47.2 ± 9.0	48.2 ± 11.1	45.0 ± 9.7
HR	[bpm]	111.3 ± 14.9	109.0 ± 11.4	106.0 ± 11.2	113.5 ± 12.1	116.9 ± 14.5	116.0 ± 14.9	116.6 ± 18.3	116.7 ± 21.0

The PaO_2_ was stable and did not change significantly in the tested conditions. The temperature of the animal was reported to be statistically different in the first steps of pH change without dead space.

The pattern of ventilation (see online resource: supplementary Table 2) was characterized by statistically significant different values of V_M_ between pH 7.40 vs. 7.25 and between pH 7.35 vs. 7.25 (both without dead space). No statistically detectable change was found during the application of dead space. Noteworthy, comparing V_M_ during ventilation with and without dead space, at pH 7.40, 7.35, 7.30, a statistically significant difference was found.

The V_T_ was characterized by a significant increase of its values when decreasing pH in the two different phases of the experiment (see Fig. 4) and this difference became statistically significant in different steps of this rise (see online resource: supplementary Table 2).

The application of dead space brought a consistent increase in V_T_ when compared with measurements performed without it.

Respiratory rate did not change significantly when no dead space was applied. However, with the application of dead space, RR reduced in a statistically significant fashion when applying the dead space (with exception when passing between 7.30 and 7.25). Moreover comparing the RR, at the same pH level and differing for the application of the dead space, these last showed statistically significant lower values with the exception at pH 7.40.

For the other hemodynamic parameters, substantial stability was observed (see Table 1) and when a statistically significant difference was found (Table 1), its change had limited magnitude.

### Artificial neural network

**Fig. 5:**
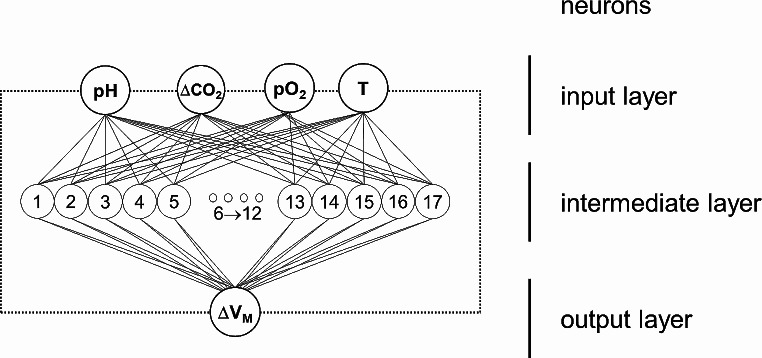
The implemented Artificial Neural Network

The ANN architecture showing the best performance on the assigned task during the first phase of the study was the one with 17 intermediate neurons (Fig. 5) which showed an MSE of 0.004 and an R^2^ of 0.998.

The best ANN was identified among the 20 trained with 17 intermediate neurons; it had an MSE of 0.001 and an R^2^ of 0.999 and it was picked for the prospective test.

**Fig. 6:**
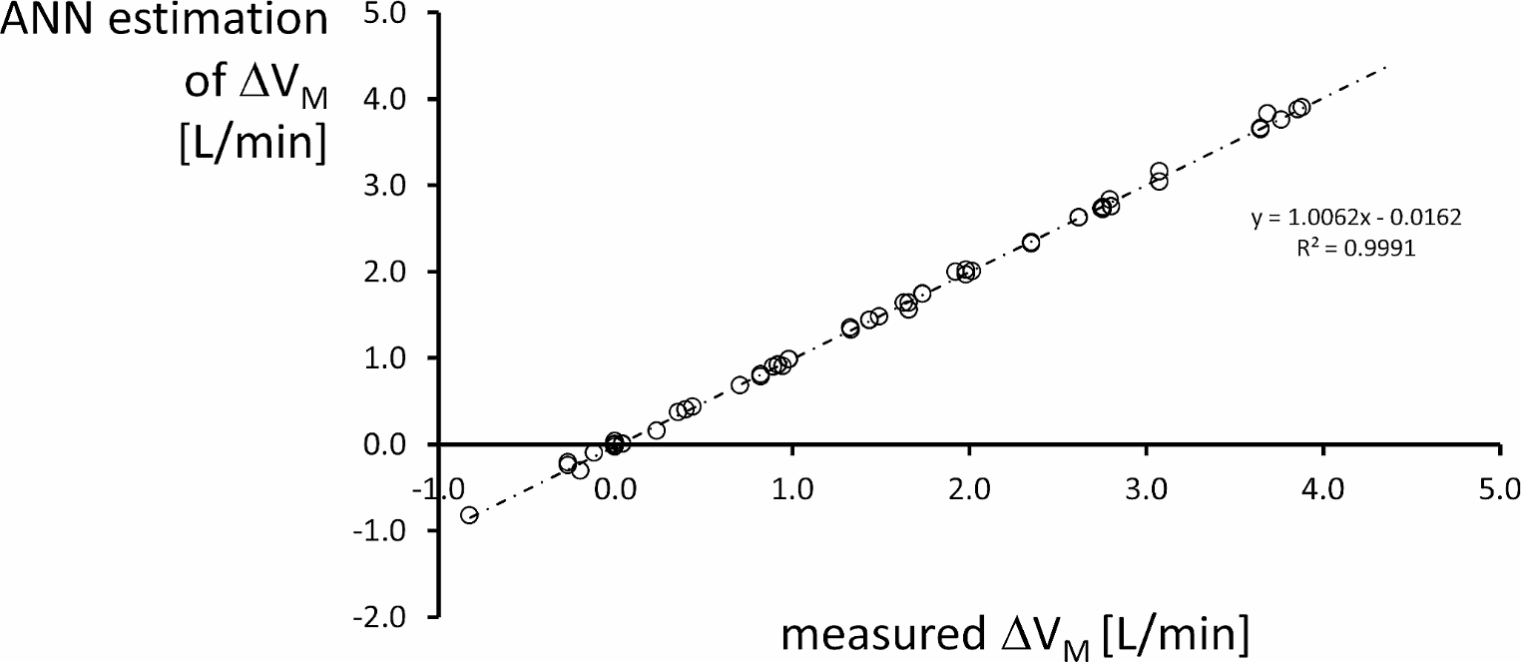
Regression between estimation of ΔV_M_ by the artificial neural network (ANN) and the effectively measured value. The graph reports also the regression equation and the relative R^2^

After testing it with the set of data that had not been seen before (15% of the entire pool), the ANN’s ability to assess ΔV_M_ was expressed by the linear regression *y* = 1.006*x* – 0.0162 and R^2^ = 0.999 (where *x* represents measured physiological data and *y* is the output of the ANN). See Fig. 6.

**Fig. 7:**
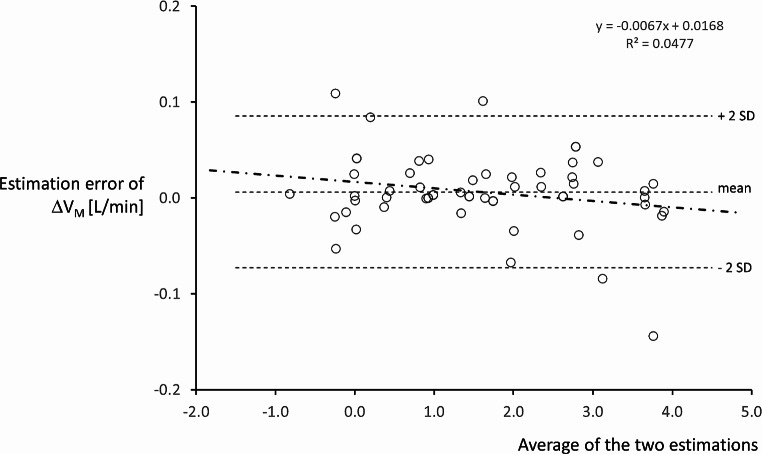
Bland-Altman analysis. The estimation error of ΔV_M_ (difference between the computation by the ANN and the effectively measured value) is plotted versus the average of the two estimations. The three dotted lines represent the mean and two standard deviations of the estimation error. The dash-dot line is the regression line between the estimation error and the average of the two estimations; its corresponding regression equation is reported above the graph. Details in the text

Analysis of the estimation error (between the ANN and measured physiological data of ΔV_M_) according to Bland and Altman displayed a bias ± SD of 0.006 ± 0.039 [L/min]. (See Fig. 7). The regression line of the estimation error vs. the average of the two measurements was expressed by y = 0.0067x + 0.016 with *R* = 0.047.

## Discussion

This study is composed of two parts. One physiological experiment provided a map of the correspondence between metabolic acidosis and respiratory output, including the perturbation caused by a dead space. The second part of the study used the data of the physiological model to train an artificial neural network to replicate the output from the respiratory centers.

### Physiological experiment

Recording the physiological changes during the administration of lactic acid and the application of dead space confirms the findings of classical physiology regarding the PaCO_2_ and the pattern of breathing [13]. The infusion of lactic acid induced a metabolic acidosis and consequently, the body’s homeostatic mechanisms attempted to establish a respiratory compensation by increasing the V_M_. As also expected, the application of dead space shifts the PaCO_2_ to higher levels, due to the rebreathing of CO_2_ inside the respiratory circuit. The physiological effects of the application of an external dead space are well-known [14] and constitute a reliable method to explore the compensatory mechanisms by the respiratory centers, even in clinical studies [15] .

It is interesting to observe in Fig. 4 that the main mechanism for V_M_ increase during dead space application is based on the increase in V_T_, while the respiratory frequency remains stable or even decreases. Also, this result does not come unexpectedly, being confirmed by classical experimental observations [16].

In general, the simplest relation between PaCO_2_ and V_M_ is expressed by an inverse proportionality, graphically represented by the so-called “metabolic hyperbole” [17] and known for many years [18]. However, these observations were performed in steady-state conditions and disregarded many factors that can modulate this response, as more recent studies on the neural control of breathing have demonstrated [2]. In fact, not only the PaO_2_ plays an already demonstrated role [19] but also the body temperature can modify the relationship between respiratory output and blood gases.

This argument implies that the complexity of respiratory control is high, as ascertained in the physiological literature [20].

We have used the change in arterial partial pressure of CO_2_ from its initial value at the beginning of the experiment (ΔPaCO_2_) and not its absolute value because, in physiological conditions, this value can be slightly different in relation to the interplay between CO_2_ production due to metabolism [21], CO_2_ body stores [22], and the efficiency of its removal. On this last point, respiratory fatigue also plays a role.

The relation between energy expenditure and respiratory frequency is composed of a resistive and elastic component and has altogether a “U-shape” [4]. The respiratory muscles, during the attempt to establish a higher minute volume by increasing the V_T_ and/or the RR, may develop muscle fatigue [22], also because the energy expenditure per breath becomes higher than in steady-state conditions. Moreover, in our experiment, not only the respiratory pattern could become more challenging for the animals (because of metabolic acidosis), but also the addition of dead space, increasing the resistive component of the work of breathing, heightened the energy expenditure and, consequently, the potential for fatigue.

For these reasons, we chose a cross-over design: the animals were randomized for receiving the application of dead space during the first or the second half of the experiment (see Fig. 2). In this way, we could avoid adding a resistive load only to the second phase of the experiment.

The first and the second parts of the experiment were separated by the administration of Tribonat, which helped to neutralize the excess acid equivalents [23] due to the administration of lactic acid and to reset the acid-base status of the animal.

As reported in the result section, it is worth noting that not all the step change variations of the collected physiological variables reached the level of statistically significant difference: this was an effect of the complexity of the model, the small sample size, and the small step changes between the imposed pHs.

However, the description of physiological alterations during acidosis was not the main goal of the experiment, having these mechanisms already been previously elucidated [24]. The main aim of the first part of the experiment was to produce the pool of examples on which the training of the ANN could be performed. Acknowledging the determinant role of pH, ΔPaCO_2_, PaO_2,_ and T, these were used as input for the implemented ANN.

### ANN experiment

The executed physiological experiment provided the number of examples necessary to train an artificial neural network. Using this data set, it was possible to identify the best architecture for the desired task and train it.

The final ANN was trained successfully with a good correlation (R^2^ = 0.999) and a low bias of 0.006 ± 0.039 [L/min].

This study has been planned to constitute a proof of concept that is possible to reproduce the response of the control of respiration by using artificial neural networks in one of their most simple configurations, the so-called multilayer perceptron. This kind of architecture has been defined as a universal function approximator [8]. They differ from the so-called architectures for deep learning mainly by the number of intermediate layers and are, for this reason, commonly labeled as *shallow neural networks*.

Ideally, to create proper examples for the training, one should design a physiology experiment to present all possible combinations of respiratory parameters. Instead of passively assembling data patterns deriving from observation of physiological conditions, a possible way to proceed is to execute biological experiments where the system is perturbated to elicit behaviors difficult to find in normal practice. In the case of the present study, the change of pH in a controlled way allowed us to record the relationship between the respiratory centers and metabolic acidosis.

A common question regarding using artificial neural networks to replicate respiratory centers’ activity is why use them when there are already analytical models of their functioning [25]. Although analytical models are important tools for understanding respiratory function, using these models in a clinical setting can be challenging. This is due to the need to assign values to multiple parameters and, more importantly, to determine how to translate physiological information into a ventilatory strategy that effectively supports a failing respiratory function.

The answer to the question lies in the purpose of the study or, using other words, which is the property of the measures we aim to reach. When the idea is to explore the possibility of controlling a device by the output of a signal analysis tool, the inherent robustness and noise immunity of an ANN [26] is particularly useful.

The idea that the future of mechanical ventilation goes through methods of closed-loop assistance has been expressed many years ago [27] and revised quite recently [28]. Our group has previously investigated the possible estimation of respiratory parameters from ventilatory tracings by using ANN [29–31] and approached the evaluation of their robustness [32]. In 2004, Chatburn [33] and Perchiazzi [34] mentioned the use of artificial neural networks as a future tool to control respiratory support. However, their effective implementation in this usage is still pending, although closed-loop mechanical ventilation has moved different steps forward [7], mainly relying upon *white-box* approaches [35]. It is important to underline that the main aim of this contribution was not to model the many interactions between the respiratory centers, which per se is a quite complex task [20]. Our goal is only to reproduce their behavior when they are exposed to a certain pattern of arterial blood gas that can be found during clinical practice in the intensive care unit.

### Limitations and perspectives

We have tested one type of ANN, falling in the broad family of multilayer perceptrons. We have not tested ANN characterized by multiple intermediate layers. So, we cannot exclude that the results of the estimation of the output of the respiratory system could have been even better using this other ANN structure. The same can be said about the learning algorithm and its parameters.

The studied ANN associates the ΔV_M_ with the ABG pattern and does not base its answer on the combination of RR and V_T_. Since these two variables are relevant for implementing computer-aided tools for mechanical ventilation, future studies should address the possibility that ANNs also yield this partition.

A similar discussion should be done about the characteristics of the sample used for the training and the testing. As with all data-driven technologies, the dependency of proper learning on the representativity of the sample is well known. On the other hand, the present experiment has been designed for creating the data base of examples for feeding the ANN and overcoming the limitations of a passive collection of tracings. This means that it is not possible to infer the performance of the trained ANN on other pathophysiological conditions not presented to it during the training. The experiment reported in this contribution is proof of concept of a possible way to proceed with gathering data on specific pathophysiological conditions.

These experiments tested the ANN technology on a limited number of conditions, including external dead space and pH changes induced by lactate infusion: future studies are required to determine its performance in various other settings, such as assisted ventilation or different pathological conditions.

It is worth mentioning that ketamine has a lower impact on respiratory drive when compared to other general anesthetics for continuous infusion. This does not exclude that a continuous infusion of ketamine modifies the output from the respiratory drive. In other words, the trained ANN has learned the functioning of the brainstem during a specific pharmacologic stimulus. This warrants further studies for training ANN to reproduce the function of the brainstem in the absence of sedation.

In this context, it would be remarkable to assess whether neural networks’ performance remains unchanged even with more extreme alterations in respiratory physiology.

Despite this, due to its remarkable performance and unique characteristics, ANN technology would be an essential tool in developing a system that interfaces information from sensors to ventilators in a closed-loop fashion in the ICU.

## Electronic supplementary material

Below is the link to the electronic supplementary material.

Supplementary Material 1

## Data Availability

The authors declare that the data supporting the findings of this study are available within the paper and its Supplementary Information files. Should any raw data files be needed, they are available from the corresponding author upon reasonable request.

## Declarations

### Statement on artificial intelligence

No method of creative artificial intelligence was used to generate the text or the figures of the present report.
