# Supplementary material for: Imitating the respiratory activity of the brain stem by using artificial neural networks: exploratory study on an animal model of lactic acidosis and proof of concept
Source: J Clin Monit Comput. 2024 Aug 20;38(6):1269–80. doi: 10.1007/s10877-024-01208-4 (PMC11604730; doi:10.1007/s10877-024-01208-4)
Supplement: Supplementary file 1 — Supplementary Material 1 [file 10877_2024_1208_MOESM1_ESM.pdf]

Supplementary Material for the Journal of Clinical Monitoring and Computing

Imitating the respiratory activity of the brain stem by using artificial neural networks: exploratory study on an animal model of lactic acidosis and proof of concept

Gaetano Perchiazzi[1,2], Rafael Kawati[2], Mariangela Pellegrini[1,2], Jasmine Liangpansakul[1], Roberto Colella[3], Paolo Bollella[4], Pramod Rangaiah[5], Annamaria Cannone[6], Deepthi Hulithala Venkataramana[7], Mauricio Perez[5], Sebastiano Stramaglia[8,9], Luisa Torsi[4], Roberto Bellotti[8,9], Robin Augustine[5]

[1]The Hedenstierna Laboratory, Department of Surgical Sciences, Uppsala University, Uppsala, Sweden
[2] Department of Anesthesia, Operation and Intensive Care, Uppsala University Hospital, Uppsala, Sweden
[3] Ministry of Education and Merit, Italy
[4] Department of Chemistry, University of Bari Aldo Moro, Bari, Italy.
[5] Department of Electrical Engineering, Solid-State Electronics, Uppsala University, Uppsala, Sweden
[6] Department of Anaesthesia and Intensive Care, "Madonna delle Grazie" Hospital, Matera, Italy
[7] Department of Information Technology, Uppsala University, Uppsala, Sweden
[8] Dipartimento Interateneo di Fisica, Università degli Studi di Bari, Italy.
[9] Istituto Nazionale di Fisica Nucleare, Sezione di Bari, Italy.

Running Head: ANN and respiration

Corresponding author:
Gaetano Perchiazzi, MD, PhD
Hedenstierna Laboratoriet, Akademiska sjukhuset ing 40 3 tr,
75185 Uppsala, Sweden
e-mail **gaetano.perchiazzi@uu.se**

Caption for Online Resources - supplementary tables 1A, 1B, 1C, 1D.

The tables summarize the statistical tests performed on the measured variables, between groups characterized by different levels of imposed metabolic acidosis.

White circles label the absence of dead space, black circles indicate the presence of dead space.

p indicates the probability of accepting the null hypothesis that the two groups are equal according to Wilcoxon test.

a/n shows the cut-off for declaring a statistical difference according to Bonferroni method.

Asterisks '*' mark a statistically significant difference between the tested groups.

Abbreviations: $PaCO_2$: partial pressure of carbon dioxide in arterial blood; PaO_2: partial pressure of oxygen in arterial blood; HCO^-_3 : bicarbonate ion; Temp: temperature; BE: base excess; SaO_2 : oxygen saturation in arterial blood; V_M: minute volume; V_T : tidal volume; RR: respiratory rate; Syst Sys: systemic systolic arterial pressure; Dia Sys: systemic diastolic arterial pressure; Syst Pol: pulmonary systolic arterial pressure; Dia Pol: pulmonary diastolic arterial pressure; CO: cardiac output; Wedge: pulmonary capillary wedge pressure; HR: heart rate. E_TCO_2 : end-tidal carbon dioxide. Letters J, K, L, and M correspond to imposed pH of 7.40, 7.35, 7.30, and 7.25, respectively.

Supplementary table 1A

measured variable		○ = no dead space<br>● = dead space	p	α/n	statistical significance
pH	J vs K	○	0.0020	0.017	*
	J vs L	○	0.0020	0.017	*
	J vs M	○	0.0039	0.017	*
	K vs L	○	0.0020	0.017	*
	K vs M	○	0.0039	0.017	*
	L vs M	○	0.0039	0.017	*
	J vs K	●	0.0020	0.017	*
	J vs L	●	0.0020	0.017	*
	J vs M	●	0.0020	0.017	*
	K vs L	●	0.0020	0.017	*
	K vs M	●	0.0020	0.017	*
	L vs M	●	0.0020	0.017	*
	J	○ vs ●	1.0000	0.050	
	K	○ vs ●	1.0000	0.050	
	L	○ vs ●	1.0000	0.050	
	M	○ vs ●	0.2500	0.050	
PaCO2	J vs K	○	0.6250	0.017	
	J vs L	○	0.9219	0.017	
	J vs M	○	0.8203	0.017	
	K vs L	○	0.4316	0.017	
	K vs M	○	0.0234	0.017	
	L vs M	○	0.1719	0.017	
	J vs K	●	0.0273	0.017	
	J vs L	●	0.0195	0.017	
	J vs M	●	0.0098	0.017	*
	K vs L	●	0.0371	0.017	
	K vs M	●	0.0020	0.017	*
	L vs M	●	0.0195	0.017	
	J	○ vs ●	0.1055	0.050	
	K	○ vs ●	0.0117	0.050	*
	L	○ vs ●	0.0020	0.050	*
	M	○ vs ●	0.0039	0.050	*
PaO2	J vs K	○	0.4492	0.017	
	J vs L	○	1.0000	0.017	
	J vs M	○	0.8203	0.017	
	K vs L	○	0.3262	0.017	
	K vs M	○	0.2070	0.017	
	L vs M	○	0.6641	0.017	
	J vs K	●	0.0840	0.017	
	J vs L	●	0.0879	0.017	
	J vs M	●	0.0273	0.017	
	K vs L	●	0.3203	0.017	
	K vs M	●	0.6953	0.017	
	L vs M	●	0.3906	0.017	
	J	○ vs ●	0.2324	0.050	
	K	○ vs ●	0.2227	0.050	
	L	○ vs ●	0.4316	0.050	
	M	○ vs ●	0.1953	0.050	
Temp	J vs K	○	0.0313	0.017	
	J vs L	○	0.0078	0.017	*
	J vs M	○	0.0039	0.017	*
	K vs L	○	0.0625	0.017	
	K vs M	○	0.0039	0.017	*
	L vs M	○	0.0625	0.017	
	J vs K	●	0.7500	0.017	
	J vs L	●	0.0742	0.017	
	J vs M	●	0.0234	0.017	
	K vs L	●	0.0625	0.017	
	K vs M	●	0.0313	0.017	
	L vs M	●	0.1094	0.017	
	J	○ vs ●	0.2676	0.050	
	K	○ vs ●	0.8457	0.050	
	L	○ vs ●	0.5508	0.050	
	M	○ vs ●	0.6172	0.050	

Supplementary table 1B

measured variable		○ = no dead space<br>● = dead space	p	α/n	statistical significance
**HCO3**	J vs K	○	0.0039	0.017	*
	J vs L	○	0.0020	0.017	*
	J vs M	○	0.0039	0.017	*
	K vs L	○	0.0020	0.017	*
	K vs M	○	0.0039	0.017	*
	L vs M	○	0.0039	0.017	*
	J vs K	●	0.0098	0.017	*
	J vs L	●	0.0020	0.017	*
	J vs M	●	0.0020	0.017	*
	K vs L	●	0.0020	0.017	*
	K vs M	●	0.0020	0.017	*
	L vs M	●	0.0020	0.017	*
	J	○ vs ●	0.0840	0.050	
	K	○ vs ●	0.0273	0.050	*
	L	○ vs ●	0.0020	0.050	*
	M	○ vs ●	0.0039	0.050	*
**BE**	J vs K	○	0.0020	0.017	*
	J vs L	○	0.0020	0.017	*
	J vs M	○	0.0039	0.017	*
	K vs L	○	0.0020	0.017	*
	K vs M	○	0.0039	0.017	*
	L vs M	○	0.0039	0.017	*
	J vs K	●	0.0020	0.017	*
	J vs L	●	0.0020	0.017	*
	J vs M	●	0.0020	0.017	*
	K vs L	●	0.0020	0.017	*
	K vs M	●	0.0020	0.017	*
	L vs M	●	0.0020	0.017	*
	J	○ vs ●	0.0840	0.050	
	K	○ vs ●	0.0371	0.050	*
	L	○ vs ●	0.0020	0.050	*
	M	○ vs ●	0.0039	0.050	*
**VM**	J vs K	○	0.1484	0.017	
	J vs L	○	0.0547	0.017	
	J vs M	○	0.0156	0.017	*
	K vs L	○	0.3750	0.017	
	K vs M	○	0.0039	0.017	*
	L vs M	○	0.0547	0.017	
	J vs K	●	0.2754	0.017	
	J vs L	●	0.1309	0.017	
	J vs M	●	0.0371	0.017	
	K vs L	●	0.3223	0.017	
	K vs M	●	0.3223	0.017	
	L vs M	●	0.9219	0.017	
	J	○ vs ●	0.0156	0.050	*
	K	○ vs ●	0.0059	0.050	*
	L	○ vs ●	0.0098	0.050	*
	M	○ vs ●	0.0547	0.050	
**VT**	J vs K	○	0.0078	0.017	*
	J vs L	○	0.0078	0.017	*
	J vs M	○	0.0078	0.017	*
	K vs L	○	0.0273	0.017	
	K vs M	○	0.0039	0.017	*
	L vs M	○	0.1289	0.017	
	J vs K	●	0.0371	0.017	
	J vs L	●	0.0098	0.017	*
	J vs M	●	0.0020	0.017	*
	K vs L	●	0.0195	0.017	
	K vs M	●	0.0039	0.017	*
	L vs M	●	0.0488	0.017	
	J	○ vs ●	0.0156	0.050	*
	K	○ vs ●	0.0020	0.050	*
	L	○ vs ●	0.0039	0.050	*
	M	○ vs ●	0.0039	0.050	*

Supplementary table 1C

measured variable		○ = no dead space<br>● = dead space	p	α/n	statistical significance
**RR**	J vs K	○	0.2500	0.017	
	J vs L	○	0.4609	0.017	
	J vs M	○	0.7422	0.017	
	K vs L	○	0.1934	0.017	
	K vs M	○	0.3008	0.017	
	L vs M	○	0.6523	0.017	
	J vs K	●	0.0098	0.017	*
	J vs L	●	0.0098	0.017	*
	J vs M	●	0.0039	0.017	*
	K vs L	●	0.1309	0.017	
	K vs M	●	0.0098	0.017	*
	L vs M	●	0.0098	0.017	*
	J	○ vs ●	0.5469	0.050	
	K	○ vs ●	0.0059	0.050	*
	L	○ vs ●	0.0137	0.050	*
	M	○ vs ●	0.0039	0.050	*
**Syst Sys**	J vs K	○	0.5430	0.017	
	J vs L	○	0.1289	0.017	
	J vs M	○	0.4180	0.017	
	K vs L	○	0.3340	0.017	
	K vs M	○	0.8438	0.017	
	L vs M	○	0.9219	0.017	
	J vs K	●	0.2793	0.017	
	J vs L	●	0.7422	0.017	
	J vs M	●	0.8438	0.017	
	K vs L	●	0.2402	0.017	
	K vs M	●	0.3848	0.017	
	L vs M	●	0.8047	0.017	
	J	○ vs ●	0.7383	0.050	
	K	○ vs ●	0.3125	0.050	
	L	○ vs ●	0.4766	0.050	
	M	○ vs ●	0.9375	0.050	
**Dia Sys**	J vs K	○	0.3809	0.017	
	J vs L	○	0.5098	0.017	
	J vs M	○	0.2031	0.017	
	K vs L	○	0.9844	0.017	
	K vs M	○	0.1719	0.017	
	L vs M	○	0.2500	0.017	
	J vs K	●	1.0000	0.017	
	J vs L	●	0.0703	0.017	
	J vs M	●	0.2383	0.017	
	K vs L	●	0.1934	0.017	
	K vs M	●	0.4102	0.017	
	L vs M	●	0.5625	0.017	
	J	○ vs ●	0.0332	0.050	*
	K	○ vs ●	0.0254	0.050	*
	L	○ vs ●	0.9453	0.050	
	M	○ vs ●	0.3750	0.050	
**Sys Pol**	J vs K	○	0.3906	0.017	
	J vs L	○	0.0703	0.017	
	J vs M	○	0.0586	0.017	
	K vs L	○	0.0078	0.017	*
	K vs M	○	0.0078	0.017	*
	L vs M	○	0.1758	0.017	
	J vs K	●	0.8203	0.017	
	J vs L	●	0.6523	0.017	
	J vs M	●	1.0000	0.017	
	K vs L	●	0.9219	0.017	
	K vs M	●	0.9766	0.017	
	L vs M	●	0.3828	0.017	
	J	○ vs ●	0.3457	0.050	
	K	○ vs ●	0.6074	0.050	
	L	○ vs ●	0.2715	0.050	
	M	○ vs ●	0.3711	0.050	

Supplementary table 1D

measured variable		○ = no dead space<br>● = dead space	p	α/n	statistical significance
Dia Pol	J vs K	○	0.2656	0.017	
	J vs L	○	0.9805	0.017	
	J vs M	○	0.3125	0.017	
	K vs L	○	0.9375	0.017	
	K vs M	○	0.5547	0.017	
	L vs M	○	0.6523	0.017	
	J vs K	●	0.2383	0.017	
	J vs L	●	0.9023	0.017	
	J vs M	●	0.3984	0.017	
	K vs L	●	0.3750	0.017	
	K vs M	●	0.7773	0.017	
	L vs M	●	0.5234	0.017	
	J	○ vs ●	0.3789	0.050	
	K	○ vs ●	0.3125	0.050	
	L	○ vs ●	0.6914	0.050	
	M	○ vs ●	0.4766	0.050	
CO	J vs K	○	0.5703	0.017	
	J vs L	○	0.1289	0.017	
	J vs M	○	0.0391	0.017	
	K vs L	○	0.0391	0.017	
	K vs M	○	0.0391	0.017	
	L vs M	○	0.0156	0.017	*
	J vs K	●	0.0391	0.017	
	J vs L	●	0.0977	0.017	
	J vs M	●	0.3711	0.017	
	K vs L	●	0.1484	0.017	
	K vs M	●	0.0039	0.017	*
	L vs M	●	0.0117	0.017	*
	J	○ vs ●	0.0664	0.050	
	K	○ vs ●	0.4961	0.050	
	L	○ vs ●	0.9102	0.050	
	M	○ vs ●	0.3516	0.050	
Wedge	J vs K	○	0.5078	0.017	
	J vs L	○	0.4219	0.017	
	J vs M	○	0.2188	0.017	
	K vs L	○	0.6563	0.017	
	K vs M	○	0.3594	0.017	
	L vs M	○	0.3125	0.017	
	J vs K	●	0.7891	0.017	
	J vs L	●	0.1719	0.017	
	J vs M	●	0.7344	0.017	
	K vs L	●	0.2500	0.017	
	K vs M	●	0.7500	0.017	
	L vs M	●	0.3594	0.017	
	J	○ vs ●	0.6563	0.050	
	K	○ vs ●	0.3828	0.050	
	L	○ vs ●	0.0781	0.050	
	M	○ vs ●	1.0000	0.050	
ETCO2	J vs K	○	0.4688	0.017	
	J vs L	○	0.9336	0.017	
	J vs M	○	0.2188	0.017	
	K vs L	○	0.8281	0.017	
	K vs M	○	0.4219	0.017	
	L vs M	○	0.2031	0.017	
	J vs K	●	0.0703	0.017	
	J vs L	●	0.0820	0.017	
	J vs M	●	0.4063	0.017	
	K vs L	●	0.5313	0.017	
	K vs M	●	0.1484	0.017	
	L vs M	●	0.2266	0.017	
	J	○ vs ●	0.0742	0.050	
	K	○ vs ●	0.0195	0.050	*
	L	○ vs ●	0.0547	0.050	
	M	○ vs ●	0.0313	0.050	*
HR	J vs K	○	0.6875	0.017	
	J vs L	○	0.1719	0.017	
	J vs M	○	0.4375	0.017	
	K vs L	○	0.4844	0.017	
	K vs M	○	0.0625	0.017	
	L vs M	○	0.0625	0.017	
	J vs K	●	1.0000	0.017	
	J vs L	●	1.0000	0.017	
	J vs M	●	0.9688	0.017	
	K vs L	●	0.8125	0.017	
	K vs M	●	0.9063	0.017	
	L vs M	●	1.0000	0.017	
	J	○ vs ●	0.3125	0.050	
	K	○ vs ●	0.1094	0.050	
	L	○ vs ●	0.1563	0.050	
	M	○ vs ●	0.8438	0.050	

Supplementary Table 2

			Vm	VT	RR
respiratory pattern	J vs K	○	0	*	0
	J vs L	○	0	*	0
	J vs M	○	*	*	0
	K vs L	○	0	0	0
	K vs M	○	*	*	0
	L vs M	○	0	0	0
	J vs K	●	0	0	*
	J vs L	●	0	*	*
	J vs M	●	0	*	*
	K vs L	●	0	0	0
	K vs M	●	0	*	*
	L vs M	●	0	0	*
	J	○ vs ●	*	*	0
	K	○ vs ●	*	*	*
	L	○ vs ●	*	*	*
	M	○ vs ●	0	*	*

Caption for Online Resource - supplementary table 2.
Summarizes the statistical tests performed on the respiratory variables, between groups characterized by different levels of imposed metabolic acidosis. White circles label the absence of dead space, black circles indicate the presence of dead space. Asterisks '*' mark a statistically significant difference between the tested groups.
Abbreviations: V_M: minute volume; V_T : tidal volume; RR: respiratory rate; Letters J, K, L, and M correspond to imposed pH of 7.40, 7.35, 7.30, and 7.25, respectively.
